# Inhibiting Microglia-Derived NLRP3 Alleviates Subependymal Edema and Cognitive Dysfunction in Posthemorrhagic Hydrocephalus after Intracerebral Hemorrhage via AMPK/Beclin-1 Pathway

**DOI:** 10.1155/2022/4177317

**Published:** 2022-05-17

**Authors:** Zhaoqi Zhang, Peiwen Guo, Suna Huang, Zhengcai Jia, Tunan Chen, Xin Liu, Hua Feng, Yujie Chen

**Affiliations:** ^1^Department of Neurosurgery and State Key Laboratory of Trauma, Burn and Combined Injury, Southwest Hospital, Third Military Medical University (Army Medical University), Chongqing 400038, China; ^2^Chongqing Key Laboratory of Precision Neuromedicine and Neuroregenaration, Southwest Hospital, Third Military Medical University (Army Medical University), Chongqing 400038, China; ^3^Chongqing Clinical Research Center for Neurosurgery, Southwest Hospital, Third Military Medical University (Army Medical University), Chongqing 400038, China; ^4^CAS Key Laboratory of Separation Science for Analytical Chemistry, Dalian Institute of Chemical Physics, Chinese Academy of Sciences, Dalian 116023, China

## Abstract

For posthemorrhagic hydrocephalus (PHH) patients, whether occur subependymal edema indicates poor outcomes, partially manifested as cognitive impairment. In the brain, NLRP3 inflammasome mainly derived from microglia/macrophages is involved in proinflammatory and neurodeficits after hemorrhage, and autophagy is vital for neuronal homeostasis and functions. Accumulating evidence suggest that NLRP3 inflammasome and autophagy played an essential role after intracerebral hemorrhage (ICH). We aimed to dissect the mechanisms underlying subependymal edema formation and cognitive dysfunction. Here, based on the hydrocephalus secondary to ICH break into ventricular (ICH-IVH) in rats, this study investigated whether microglia/macrophage-derived NLRP3 induced subependymal edema formation and neuron apoptosis in subventricular zones (SVZ). In the acute phase of ICH-IVH, both the expression of NLRP3 inflammasome of microglia/macrophages and the autophagy of neurons were upregulated. The activated NLRP3 in microglia/macrophages promoted the release of IL-1beta to extracellular, which contributed to excessive autophagy, leading to neurons apoptosis both in vivo and in vitro through the AMPK/Beclin-1 pathway combined with transcriptomics. Administration of MCC950 (NLRP3 inflammasome specific inhibitor) after ICH-IVH significantly reduced edema formation and improved cognitive dysfunction. Thus, inhibiting NLRP3 activation in SVZ may be a promising therapeutic strategy for PHH patients that warrants further investigation.

## 1. Introduction

Intracerebral hemorrhage (ICH) has high morbidity and mortality and is associated with severe long-term disability [1, 2]; however, there is still no effective treatment so far [3]. About 40% of ICH break into the ventricle (ICH-IVH), and more than half of these patients will develop into varying degrees of hydrocephalus, which makes the unfavorable outcome even worse [2, 4, 5]. Although there are many hypotheses about the pathogenesis of hydrocephalus after ICH-IVH, such as blood-clot blockage, barrier impairment, inflammation, and blood components [6], due to the rare experimental verification, the exact pathogenesis remains unclear.

Clinically, the subependymal edema of imaging results is associated strongly with poor outcomes in PHH patients. Clinical treatments of reducing hydrocephalus by shunting operation or dehydrating agents have not demonstrated therapeutic efficacy in reducing subependymal edema [4, 5]. ICH-IVH causes primary brain injury through direct mechanical effects of the hematoma and leads to the development of subependymal edema as ICP increases or inflammation activation, which induces secondary brain injury manifested by loss of neurons [7, 8]. However, how the subependymal edema occurred remains unclear, especially the source of water that contributed to edema formation. As such, PHH remains the worse compliant of hemorrhage. Considering the contribution of subependymal edema to secondary clinical deterioration, subependymal edema may represent an attractive therapeutic target in PHH.

Once ICH-IVH occurred, the robust cellular immune response rapidly activated, especially the resident neuroglia (microglia/macrophages) and recruited peripheral leukocytes were soon activated to release proinflammation cytokines, causing neuroinflammation and brain injury at acute phase [9–11]. Evidence indicates that focal inflammation contributes significantly to BBB disruption and brain edema. The NLRP3 inflammasome, highly expressed in microglia/macrophages, has been proved to participate in a variety of pathological processes such as psychiatric and neurodegenerative disorders [12, 13]. Activating NLRP3 inflammasome can induce cytokines, such as IL-1beta, a new cardiovascular risk biomarker [14]. Accumulating evidence that NLRP3 inflammasome inhibition could relieve neuroinflammation, disrepute BBB intensity, and reduce cell death in early brain injury [15, 16] make the NLRP3 inflammasome-induced anti-inflammatory treatment be a potential strategy to reduce brain edema.

Autophagy is an evolutionarily conserved intracellular process to maintain cellular homeostasis by the phagosome and lysosomal pathways [17]. Abnormal autophagy has been found to play an important role in the pathogenic process of a variety of neurodegenerative diseases [18–20], such as Parkinson disease (PD) and Alzheimer disease (AD) [21, 22]. What is more, recent studies have revealed that autophagy is upregulated under some restress conditions in neurons [23], and excessive autophagy can lead to neurons apoptosis after ischemia [24]. However, there are few reports about autophagy dysfunction in the acute phase of ICH.

Herein, we explored the mechanism of subependymal edema formation-related cognitive dysfunction after ICH-IVH. Since inflammation and autophagy were closely related, we further studied the relationship between NLRP3 inflammasome (a crucial molecular regulator in inflammation) activation in microglia/macrophages and autophagy in neurons after ICH-IVH. A rat model of ICH with ventricular extension and PC12 cells were performed to address this proposal.

## 2. Materials and Methods

### 2.1. Animals

All experimental procedures were approved by the Laboratory Animal Welfare and Ethics Committee of Third Military Medical University (AMUWEC2020762) and were performed according to the guide for the care and use of laboratory animals of the national institutes of health and reported in compliance with the ARRIVE (animal research: reporting of in vivo experiments) guidelines. A total of 261 male rats (weight 220-250 g) were housed in a temperature and humidity-controlled room under a standard 12 h light/dark cycle for a minimum of 3 days before ICH-IVH induction and were provided free access to food and water.

### 2.2. ICH-IVH Model and Groups

ICH-IVH was induced by autologous arterial blood injection into the right perilateral ventricles as previously described [25]. Briefly, rats were anesthetized with pentobarbital (40 mg/kg, IP). The right femoral artery was catheterized as a source of the blood sample. A cranial burr hole (1 mm) was drilled after rats were positioned in a stereotaxic frame. Aliquots of 200 *μ*l nonheparinized arterial blood were infused into the right caudate nucleus (coordinates: 0.2 mm posterior, 2.2 mm lateral, and 5.0 mm ventral to the bregma) at a rate of 14 *μ*l/min using a microinfusion pump. The burr hole was sealed with bone wax, and skin incision was closed with sutures after the needle was removed.

Rats were randomly divided into the following 3 groups: sham, ICH-IVH, and MCC950. The sham group had only a needle insertion. MCC950 group received MCC950 (10 mg/kg, IP; MCE, USA) which was dissolved in saline at 1 hour after ICH-IVH model. ICH-IVH group was given an equal volume of saline at the same time.

### 2.3. Cell Culture and Treatment

PC12 neurons cells of rats (ScienCell, USA) were used for in vitro study. PC12 cells were cultured in Dulbecco's Modified Eagle Medium (DMEM) supplemented with 10% (*v*/*v*) fetal bovine serum (FBS) and 1% penicillin/streptomycin at 37°C in a humidified atmosphere of 5% CO_2_. After the completion of cell processing, IL-1beta (Novoprotein, China) was added to DMEM and diluted at a dosage of concentrations (10 *μ*g/l). PC12 cells were then treated with L-1*β* or Compound C for 24 h before use.

### 2.4. Apoptosis Assay

Apoptosis was detected using ANNEXIN V-FITC/PI cell apoptosis detection kit (CST, USA). PC12 cells from different groups were digested with trypsin but without EDTA, resuspended in the blinding buffer, and stained with Annexin V-FITC for 15 min and PI for 5 min. The results were analyzed by flow cytometry (Canto2, BD, USA).

### 2.5. Immunofluorescence Staining

Under deep anesthesia, rats were sacrificed by transcardial perfusion with 100 ml normal saline followed by 50 ml 4% neutral buffered paraformaldehyde. Brains were fixed in 4% neutral buffered paraformaldehyde for 24 h at 4°C followed by 25% and 30% sucrose solution until brains were dehydrated fully. Then, brains were cut into 10 *μ*m thick coronal sections using a cryostat (LM3050S, Leica, Germany) after being frozen at -80°C. Slides were washed with 0.01 M of PBS 3 times for 10 min and then incubated in 0.3% Triton X-100 for 30 min at room temperature. After being blocked with 5% BSA for 1 h at room temperature, the sections were incubated with primary antibody at 4°C overnight as follows: anti-CD31 (1: 200; Abcam, USA), anti-ZO-1 (1 : 200; Abcam, USA), anti-Caspase1 (1 : 200; NOVUS; USA), anti-IL1beta (1 : 400; GeneTex, USA), anti-Iba1 (1 : 200; Genetex, USA), anti-CD68 (1 : 200; Abcam, USA), anti-NLRP3 (1 : 200; Abcam, USA), anti-Atg5 (1 : 100; ZEN-BIO; China), anti-p62 (1 : 100; ZEN-BIO; China), and anti-NeuN (1 : 200; Abcam, USA). Then, the sections were washed with 0.01 M PBS and incubated with appropriate fluorescence-conjugated secondary antibodies (1 : 400; Invitrogen, USA) for 2 h at room temperature. The slides were observed and photographed under a fluorescence microscope (LSM880; ZEISS, Germany).

### 2.6. Western Blotting

The subventricular zone (SVZ) tissue was separated and homogenized to collect the protein samples at 3 days after hemorrhage. Equal amounts of protein samples (20 *μ*g) were loaded on SDS-PAGE gels, electrophoresed, and transferred onto a polyvinylidene difluoride membrane. The membrane was blocked and incubated overnight at 4°C with the following primary antibodies: anti-ZO-1 (1 : 1000; Abcam, USA), anti-NLRP3 (1 : 1000; Abcam, USA), anti-Caspase1 (1 : 1000; NOVUS, USA), anti-IL1beta (1 : 1000; GeneTex, USA), anti-Atg5 (1 : 1000; ZEN-BIO, China), anti-LC3B (1 : 1000; ZEN-BIO, China), anti-p62 (1 : 1000; ZEN-BIO, China), anti-pAMPK (1 : 1000; CST, USA), anti-AMPK (1 : 1000; CST, USA), anti-ULK1 (1 : 1000; CST, USA), anti-Beclin-1 (1 : 1000; CST, USA), and anti-*β*-actin (1 : 1000; CST, USA). Appropriate secondary antibodies (1 : 3000, CST; 1 : 5000, abcam) were selected to incubate with the membrane for 1 h at room temperature. The bands were probed with an ECL Plus chemiluminescence regent Kit (ThermoFisher, USA) and visualized with the image system (Bio-Rad, USA). Relative density of the protein immunoblot images was analyzed by Image J software (NIH, USA).

### 2.7. Transmission Electron Microscope

Electron microscopy was performed as previously described [26]. Rats were anaesthetised and subjected to intracardiac perfusion with 4% paraformaldehyde and 2.5% glutaraldehyde in 0.1 mol/l Sorensen's buffer (pH 7.4). The subventricular zones were removed from the brain, and a 1 mm thick coronal tissue slice was cut with a blade 4 mm overnight at 4°C. Samples were then postfixed with 1.0% OsO4 and dehydrated in graded ethyl alcohol. After dehydration was complete, the samples were infiltrated with propylene oxide, embedded in Epon resin and sectioned. Ultrathin sections were then stained with uranyl acetate and Reynold's lead citrate. Sections were evaluated using a Philips CM 100 transmission electron microscope (Hillsboro, OR, USA) and were digitally acquired using a Hamamatsu ORCA-HR camera (Hamamatsu City, Shizuoka, Japan).

### 2.8. MRI and Edema Volume Analysis

Rats were anesthesia with 2% isoflurane/air mixture throughout MRI examination. The MRI scans were performed using a 7.0-T Varian MR scanner (Bruker, USA) with a T2∗gradient-echo sequence and a T2 fasts spin-echo sequence using a view field of 35 mm∗35 mm with 20 coronal slices (1.0 mm thickness). Volumes were calculated as previously described. The edema areas were outlined, and the areas of all slices were multiplied by the section thickness [27]. All image analyses were performed using Image J (National Institutes of Health, Bethesda, Maryland, USA) by two observers in a blinded manner.

### 2.9. Brain Water Content Assessment

Brain water content was measured on day 3 after ICH-IVH, as previously described [28]. Briefly, without perfusion, the subventricular zones on both sides and cerebellum were removed. Brain tissues were weighted to measure wet weights and then dried for 24 h at 100°C to obtain dry weights. The following formula was used to calculate brain water content: (wet weight–dry weight)/wet weight × 100%.

### 2.10. TUNEL Staining

On day 3 after ICH-IVH, the brains were sampled for TUNEL staining using Apoptosis Detection Kit (Roche, USA) according to the manufacturer's instructions. The number of TUNEL-positive cells in the SVZ was counted using Image J software (NIH, USA). Six sections per brain were used for counting. Data were expressed as the number of TUNEL-positive neurons cells/mm^2^ in SVZ.

### 2.11. Fluoro-Jade C Staining

Neuronal degeneration was evaluated by Fluoro-Jade C (FJC) staining as previously reported [29]. The FJC Ready-to-Dilute Staining Kit (Biosensis Inc., Thebarton, SA, Australia) was used. Six continuous pictures of SVZ were photographed under a fluorescence microscope, and the average number of FJC-positive cells was calculated as cells/mm^2^ by Image J software.

### 2.12. Cell Counting

Cell counting was performed on brain coronal sections. Three high-power images (×40 magnification) were taken in SVZ using a digital camera. Interested positive cells were counted from 4 areas in each brain section by two researchers in a blinded manner.

### 2.13. Neurofunction Assessment

#### 2.13.1. Corner Test

On days 3, 7, and 14 after ICH-IVH, the corner turn was used to evaluate the motor and balance functions of animals as previously described [30]. In the corner turn test, each rat was allowed to proceed into a corner (the angle of 30°) for 10 times with at least 30 s intervals between every trial. The rats need to turn to right or left, and the percentage of right turns was calculated to assess the neurofunction.

#### 2.13.2. Open Field Test

Anxiety, exploratory activity, and motor function were examined in the open field test. The testing apparatus was a 100 × 100 cm square with lateral walls. A video camera suspended above recorded spontaneous motor activity over 5 min trials. Rats (*n* = 8 per group) were placed in the center of the area, and both total distances travelled and time spent in the center were recorded.

#### 2.13.3. Morris Water Maze Task

Water maze tasks were performed as described previously [31]. Briefly, rats (*n* = 8 per group) received four trials on five consecutive training days and then received a single 60 s probe trial on day 6. The latency to reach the platform during training days, the times crossing the target area (former platform position), and the time spent in the target quadrant during the probe trial were recorded.

### 2.14. H&E Staining

Rats were transcardially perfused with 0.9% sodium chloride and 4% paraformaldehyde at 3 days after ICH-IVH. The brain was dissected out after perfusion and paraffin-embedded followed by sectioning. H&E staining was performed as previously described [32].

### 2.15. Quantitative RNA Sequencing

Rats were euthanized on day 3 after ICH-IVH. RNA-Seq experiments were performed according to manufacturer's protocol, and data were analyzed by LC Biotech. Briefly, total RNA was extracted from the SVZ tissue using TRIzol reagent, and the quantified and purified total RNA were used to reverse-transcribed to generate cDNAs, which were used to synthesize U-labeled second-stranded DNAs. The ligated products were amplified with PCR, and the average inset size for the final cDNA library was 300 bp (50 bp). The expression levels of all transcripts were evaluated by calculating the fragments per kilobase per million reads. The threshold of significantly differential expression was set to *P* < 0.05 and |log_2_(fold change)| ≥ 1. The Gene Orthology (GO) and Kyoto Encyclopedia of Genes and Genomes (KEEG) databases were used to explore the biological pathways.

### 2.16. Statistical Analysis

All data were presented as mean ± SD. Data were analyzed by investigators blinded to experimental treatments. All analyses were performed using GraphPad Prism 8 (GraphPad software). We determined each sample size by power analysis using a significance level of *α* = 0.05 with 80% power to detect statistical differences. Statistical evaluation of the data was performed by analysis of variance (ANOVA), followed by Tukey multiple-comparison post hoc analysis. Statistical significance was defined as *P* < 0.05.

## 3. Results

### 3.1. NLRP3 Inhibition Attenuates Neurofunction Deficits, Especially Cognitive Dysfunction after ICH-IVH

**Figure 1:**
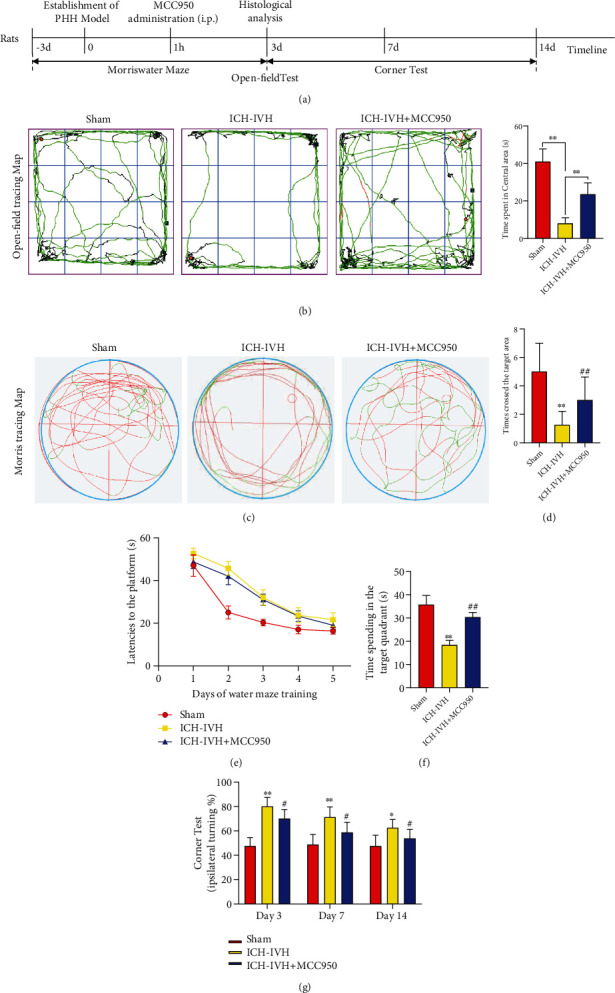
Spatial and recognition memory and spontaneous motor behavior assessment after ICH-IVH and inhibiting NLRP3 inflammasome with MCC950. (a) Schematic diagram of the experimental design. (b) Representative movement tracks and recognition memory in the sham and MCC950 treatment or not groups at 3 days after ICH-IVH. (c) Representative swim paths during the probe trial for spatial memory showing that ICH-IVH rats with MCC950 treatment made more crossings over the former platform location and spent more swim time in the target quadrant than ICH-IVH rats, indicating spatial memory impairment improved. (d) Comparison of times crossing the former target area of different groups. (e) Comparison of latency to the platform during the 5 days of Morris water maze training. (f) Comparison of time spent in the target quadrant in the probe trial. (g) Corner tests aimed at the motor function after ICH-IVH and MCC950 treatment. (*n* = 8/group). Results are presented as mean ± SD, ^∗∗^*P* < 0.01 and ^∗^*P* < 0.05 versus sham group, ^##^*P* < 0.01 and ^#^*P* < 0.05 ICH-IVH group versus MCC950 group.

According to previous studies, based on autologous blood ICH-IVH rats, we had found ICH-IVH rats showed severe motor function disorder [25]. Here, we aimed to assess cognitive dysfunction in ICH-IVH rats. To determine whether the NLRP3 inflammasome inhibitor, MCC950, affects cognitive function in ICH-IVH rats receiving MCC950 or not, we hypothesized that NLRP3 inhibition after ICH-IVH would improve cognitive function. Cognitive function was evaluated by the open-field test and the Morris test at day 3 after ICH-IVH. Compared with the sham group, we found the ICH-IVH rats had significant cognitive dysfunction and decreased exercise activity according to the open-field test results, and MCC950 treatment reduced cognitive and motor dysfunction after hemorrhage (Figure 1(b)). As for the motor dysfunction, the corner test results showed that inhibition NLRP3 improved motor deficits after ICH-IVH (Figure 1(g)). Next, the water maze test was used to evaluate cognitive function furthermore, a classical method to assess cognitive function showed the same results as the open-flied test (Figure 1(c)). There were no group differences during the training phase to find a hidden platform (Figure 1(e)). However, in the probe trial for spatial memory in which the hidden platform was removed, administration of MCC950 significantly increased the number of former platform crossings among ICH-IVH rats, and the number of former platform crossing was significantly greater among the sham group than the ICH-IVH group (Figure 1(d)). Similarly, the ICH-IVH rats with MCC950 treatment increased target quadrant time compared with the ICH-IVH group (Figure 1(f)). Collectively, these findings suggest that MCC950 administration could improve NLRP3 inflammasome-dependent neurodysfunction after ICH-IVH, especially spatial memory deficit.

### 3.2. Inhibiting NLRP3 Inflammasome Decreased Subependymal Edema after ICH-IVH

**Figure 2:**
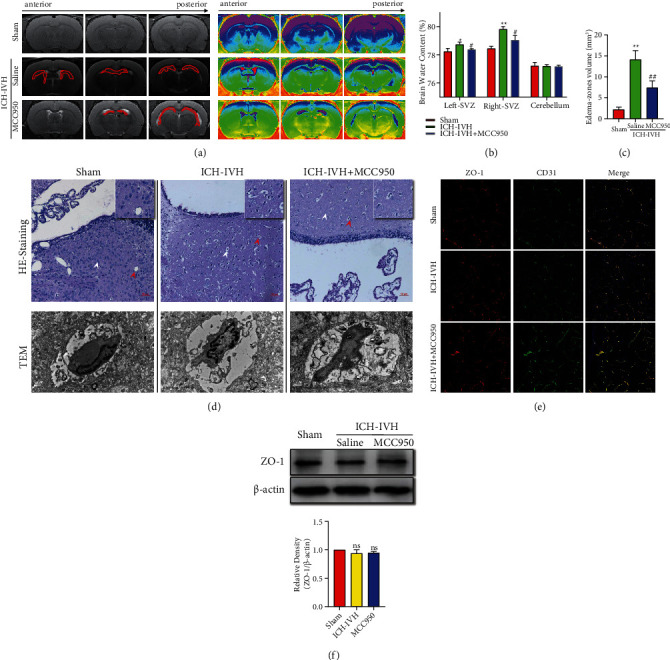
Subependymal edema assessment after ICH-IVH mediated hydrocephalus and MCC950 treatment. (a) Representative images of T2-weighted and pseudocolor showed subependymal edema at 3 days in different groups. (b) The brain water content of different parts of brain after various treatments. (c) Quantification analysis of volumes of brain edema of subventricular zones according to MRI images. (d) HE-staining images of subventricular zones tissues showed edema and capillaries conditions (upper). Bar = 50 *μ*m. TEM images of capillaries of all 3 groups (lower). Bar = 1 *μ*m. (e) Representative images of ZO-1 expressed in capillaries (CD31). Bar = 50 *μ*m. (f) Western blots images and analysis results showed the ZO-1 expression of different groups in SVZ. Results are presented as mean ± SD, ns *P* > 0.05, ^∗∗^*P* < 0.01 and ^∗^*P* < 0.05 versus sham group, ^##^*P* < 0.01 and ^#^*P* < 0.05 ICH-IVH group versus MCC950 group.

Hydrocephalus patients which had subependymal edema indicate a lousy outcome. We detected edema in subventricular zones at 3 days after ICH-IVH and MCC950 administration. According to T2 magnetic resonance imaging scans (MRIs) and pseudocolor images depending on grayscale value, we found the ICH-IVH group occurred prominent edema in SVZ. Interesting, MCC950 treatment reduced SVZ edema after ICH-IVH (Figure 2(a)). Combined with the MRIs images, we measured the edema volumes and found that inhibition NLRP3 with MCC950 decreased edema volumes after ICH-IVH at 3 days (Figure 2(c)). We also measured brain water content in different brain zones, and we found the MCC950 treatment group had lower edema than the ICH-IVH group of the subventricular zones (Figure 2(b)). Although the subventricular zones effusion had been found in many aspects, where is the source of additional water still not been explained clearly. The classical explanation of edema contained vasogenic, cellular, and osmotic brain edema [7]. Combined with HE-staining results, we found obviously interstitial edema and cellular edema after ICH-IVH. MCC950 administration reduced both two kinds of brain edema. Interestingly, the edema around capillaries was observed, and a mass of capillaries closed after ICH-IVH in SVZ. The capillaries reopened and the around edema reduced after inhibiting NLRP3 inflammasome (Figure 2(d) upper). Furthermore, observing capillaries using TEM in SVZ, we found the changes of edema around capillaries and the openness of capillaries are the same as the HE-staining results (Figure 2(d) lower). According to HE-staining and TEM results, we speculated that the edema belongs to nonangiogenic edema. To further prove this conclusion, we assessed tight junction-related protein ZO-1 of capillaries (CD31) in SVZ and found there is no difference of ZO-1 expression level among the sham group, the ICH-IVH group, and the ICH-IVH + MCC950 group (Figures 2(e) and 2(f)). Totally, the edema that occurred after hydrocephalus was nonangiogenic edema, and inhibition of NLRP3 reduced edema in SVZ.

### 3.3. MCC950 Inhibits Microglia/Macrophage-Derived NLRP3 Inflammasome Activation in SVZ

**Figure 3:**
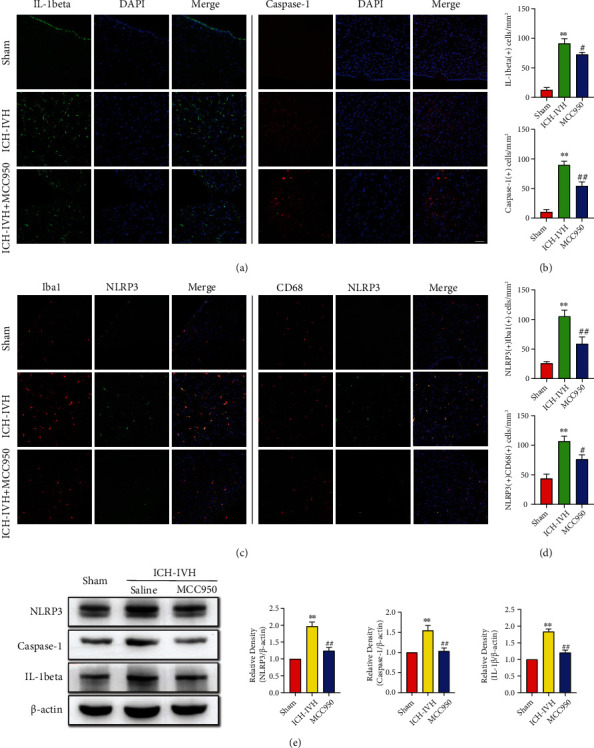
NLRP3 inflammasome and related cytokine expression of microglia/macrophages after ICH-IVH and MCC950 treatment in SVZ. (a) Representative images of IL-1beta and Caspase-1 positive cells in SVZ. Bar = 50 *μ*m. (b) Statistical results of IL-1beta and Caspase-1 positive cells in SVZ. (c) Photos of double immunofluorescence staining of NLRP3 expressed at Iba1 and CD68 positive cells in SVZ. Bar = 50 *μ*m. (d) Counting results of NLRP3-positive microglia/macrophages in SVZ. (e) Western blots images and analysis results showed the expression of NLRP3, Caspase-1, and IL-1beta with MCC950 treatment or not at 3 days after ICH-IVH. Results are presented as mean ± SD, ^∗∗^*P* < 0.01 versus sham group, ^##^*P* < 0.01 and ^#^*P* < 0.05 ICH-IVH group versus MCC950 group.

Inflammation response was activated in many kinds of diseases, leading to tissue damage and edema, especially in the acute phase. Combined with previous parts results that inhibition of NLRP3 could reduce interstitial and cellular edema, next we investigated the specific mechanism of NLRP3 inflammasome-mediated edema in SVZ. Microglia/macrophages are the main immunity and inflammation cells that react to injury or infection in the brain. The effect of MCC950 on NLRP3 inflammasome activation and IL-1beta production was examined in subventricular zones tissues of ICH-IVH rats (Figure 3(a)). At 3 days after ICH-IVH, we found IL-1beta and Caspase-1 positive cells increased in SVZ, and MCC950 treated decreased the number of IL-1beta(+) and Caspase-1(+) cells (Figure 3(b)). These indicated NLRP3-related inflammation activated in SVZ after ICH-IVH. Next, we examined the NLRP3 inflammasome expressed in which kind of cells and found NLRP3 located in microglia/macrophages after ICH-IVH. Compared with the ICH-IVH group, MCC950 treatment obviously decreased NLRP3 positive microglia/macrophages in SVZ (Figures 3(c) and 3(d)). To quantitative analysis of the expression of NLRP3 and related cytokines: IL-1beta, Caspase-1, and Western blots results showed that MCC950 treatment decreased NLRP3 inflammasome and related cytokines expression after ICH-IVH in SVZ (Figure 3(e)). We indicated that NLRP3 inflammasome activated in microglia/macrophages and released cytokines might be a reason that resulted in brain edema after ICH-IVH in SVZ.

### 3.4. NLRP3 Inhibition Prevents Neurons Excessive Autophagy-Mediated Apoptosis after ICH-IVH in SVZ

**Figure 4:**
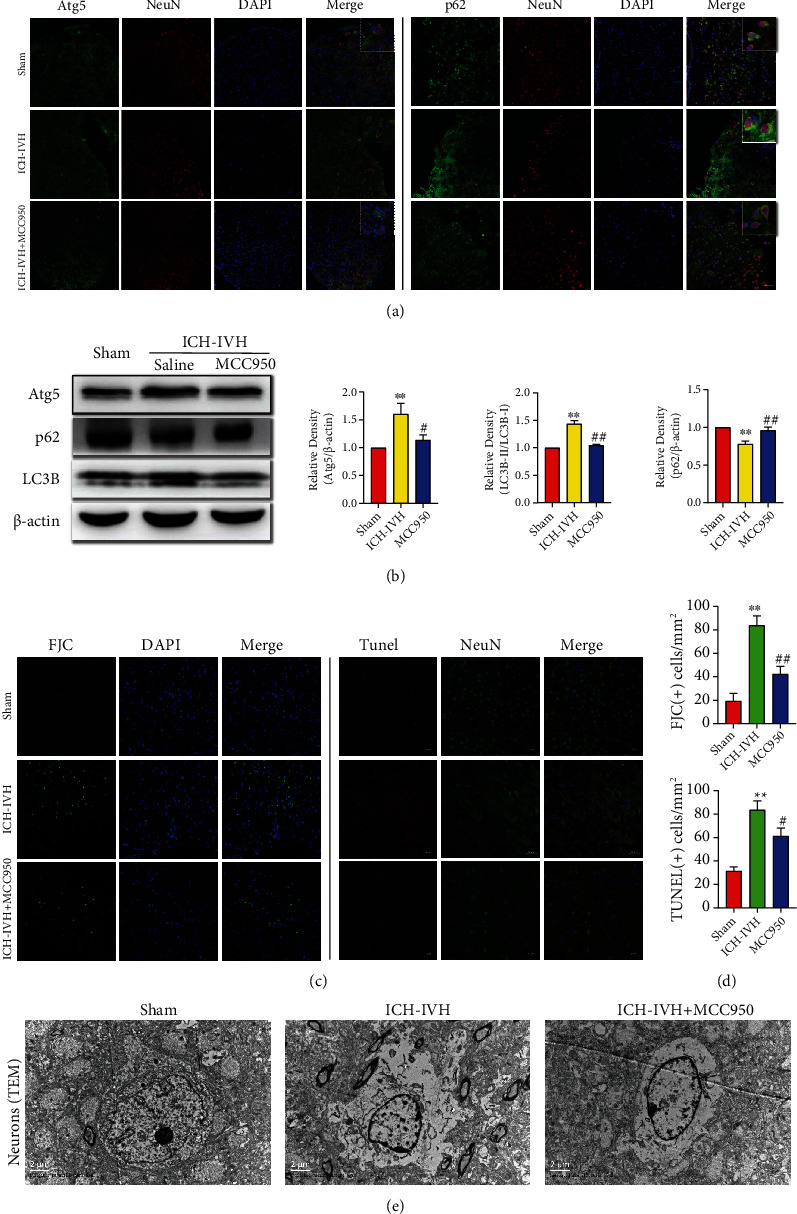
Autophagy flux changes mediated neurons degenerated and apoptosis after ICH-IVH and MCC950 treatment in SVZ. (a) Representative immunofluorescence staining images of Atg5 and p62 positive neurons in SVZ. Bar = 50 *μ*m. (b) Western blots images and analysis results showed the expression of autophagy proteins, Atg5, LC3B, and p62, in the SVZ of ICH-IVH rats receiving MCC950 or saline. (c, d) Images of FJC-staining and TUNEL-staining of neurons in SVZ after ICH-IVH and MCC950 treatment (c), analysis results of FJC(+) and TUNEL(+) neurons (d). Bar = 50 *μ*m. (e) TEM images of neurons located in SVZ showed microstructure and edema. Bar = 2 *μ*m. Results are presented as mean ± SD, ^∗∗^*P* < 0.01 versus sham group, ^##^*P* < 0.01 and ^#^*P* < 0.05 ICH-IVH group versus MCC950 group.

Subependymal edema could be improved by inhibiting NLRP3 activated in microglia/macrophages. But how the microglia/macrophage-derived edema contributed to cognitive dysfunction after ICH-IVH still needs investigation. Hence, we assessed the status of neurons in SVZ. Under normal physiological conditions, autophagy was activated at a low level to regulate cell homeostasis. After the external stimulus, autophagy is upregulated or downregulated to defence against avoiding self-injury. However, excessive autophagy out-balance led to neurons injury. We examined neurons autophagy level in SVZ and found autophagy upregulated after ICH-IVH, and administration of MCC950 reduced autophagy level in neurons. The autophagy-related proteins LC3B, Atg5 (LC3B and Atg5 indicate autophagosome formation), and p62 (p62 indicates autophagosome degradation) were detected. The images of Atg5(+) and p62(+) neurons demonstrated autophagy upregulated in neurons after ICH-IVH, and inhibited NLRP3 downregulated autophagy level of neurons (Figure 4(a)). The Western blot results examined Atg5 LC3B, and p62 expression showed the same conclusion (Figure 4(b)). Then, we evaluated the relationship between autophagy level and neuron states after ICH-IVH and MCC950 treatment; the FJC-staining and TUNEL-staining of neurons were used in this process. According to images and positive cell counting results, we found a mass of neurons dysfunction after ICH-IVH and inhibition of NLRP3 reduced FJC(+) cells and TUNEL(+) neurons in SVZ (Figures 4(c) and 4(d)). Then, we used TEM to observe neuron structure in SVZ and found neurons edema and apoptosis after ICH-IVH. After MCC950 administration, the neurons state ameliorated, and the edema also reduced (Figure 4(e)). Based on these results, we proved that inhibiting NLRP3 inflammasome expression in microglia/macrophages could reduce brain edema and downregulate neurons autophagy to protect neurological function in SVZ.

### 3.5. NLRP3 Upregulated Neuron Autophagy through the AMPK/Beclin-1 Pathway Combined with Transcriptomics

**Figure 5:**
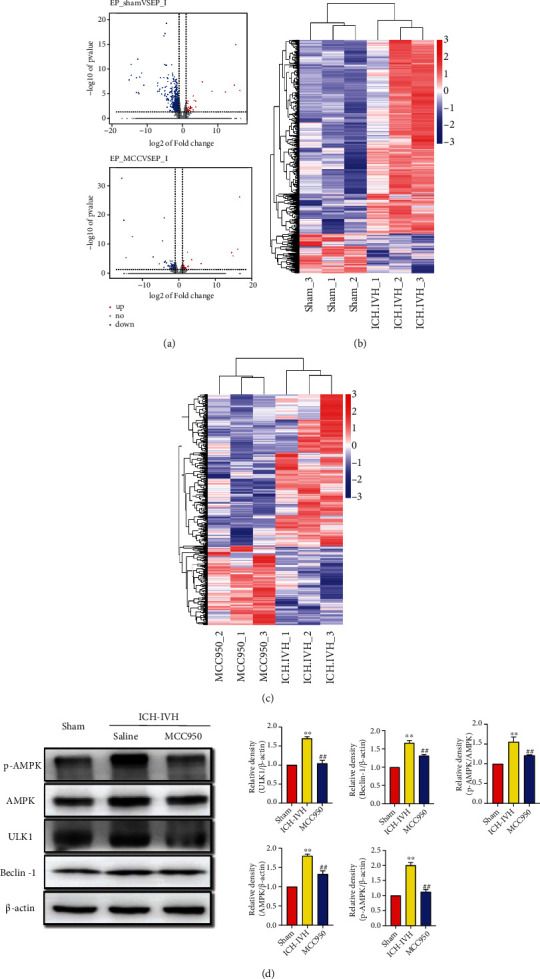
Transcriptional analysis of SVZ tissues identified AMPK/ULK1/Beclin-1 as a potential pathway of the microglia/macrophage-derived NLRP3 inflammasome and neuron excessive autophagy-mediated apoptosis after ICH-IVH. (a) Volcano plot showed differentially expressed genes in SVZ on day 3 after ICH-IVH and MCC950 treatment. (b) Heatmap of the significantly different expression genes identified by PCA for each sample between the sham group and the ICH-IVH group. (c) Heatmap showed obviously different expression genes with MCC950 treatment or not after ICH-IVH. Data were clustered hierarchically in GENE-E and colored according to row minimum and maximum. (d) Representative Western blots images of AMPK/ULK1/Beclin-1 pathway and quantitative analyses results. Data were represented as mean ± SD, ^∗∗^*P* < 0.01 versus sham group, ^##^*P* < 0.01 ICH-IVH group versus MCC950 group.

To explore the molecular mechanism between NLRP3 inflammasome and neurons autophagy, subventricular zone tissues from the sham group, the ICH-IVH group, and the ICH-IVH + MCC950 group were obtained for transcriptomics. Compared with the sham group, 565 gene expression obviously changed after ICH-IVH (518 genes upregulated, 47 genes downregulated) (Figure 5(a)). Heatmap of different expression genes after ICH-IVH contained NLRP3, autophagy, and apoptosis-related genes (Figure 5(b)). According to GO enrichment, we found the inflammatory response and innate immune response had noticeable changes (Figure S1A). Focusing on KEEG pathway enrichment, the phagosome pathway changed drastically (36 genes expression changed in this pathway) (Figure S1B). Compared with the ICH-IVH group, the MCC950 treatment group had 167 gene expression changed (Figure 5(a)). The heatmap of different expression genes after MCC950 treatment was used to select obviously related genes, which contained possible pathways that NLRP3 inflammasome mediated neuron autophagy through (Figure 5(c)). Combined with the GO enrichment pathway and KEEG enrichment pathway, the AMPK/Beclin-1 pathway was selected to explore further (Figure S1C and Figure S1D). In order to verify the transcriptomics results, Western blots were used to quantitatively analyze the expression of AMPK/Beclin-1 pathway-related protein. After ICH-IVH, the expression of AMPK, p-AMPK, ULK1, and Beclin-1 was increased. What is more, p-AMPK/AMPK was also increased. Inhibiting NLRP3 inflammasome by MCC950 downregulated the expression of essential proteins in the AMPK/Beclin-1 pathway (Figure 5(d)). Based on the above results, we speculated that the NLRP3 inflammasome might mediate neuron apoptosis through the AMPK/Beclin-1 pathway, and the AMPK/Beclin-1 pathway also could regulate autophagy. In addition, the transcriptome sequencing results also supported our previous conclusion.

### 3.6. IL-1beta Accelerated to Neurons Excessive Autophagy and Apoptosis in PC12 Cells

**Figure 6:**
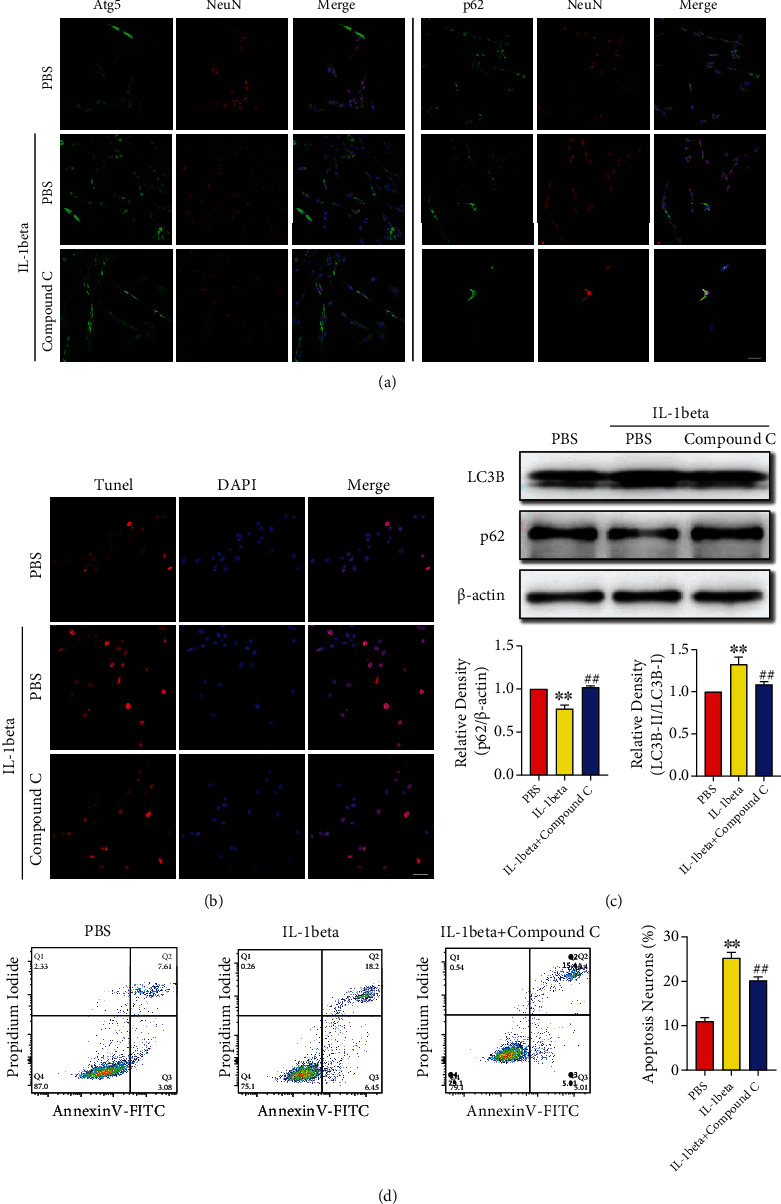
IL-1beta upregulated neurons autophagy and apoptosis, and inhibiting autophagy could reduce neurons apoptosis mediated by IL-beta which was released after NLRP3 activated. (a) Representative immunofluorescence staining images of Atg5- and p62-positive PC12 cells. Bar = 50 *μ*m. (b) Immunofluorescence TUNEL-staining images of PC12 cells, and statistic result of TUNEL(+) cells after different treatments. Bar = 50 *μ*m. (c) Representative Western blot images of LC3B and p62 expressions in PC12 cells, and statistic results of LC3B-II : LC3B-I ratio and p62 after IL-1beta and compound C treated. (d) Representative images and statistic results of flow cytometry showed early/late apoptotic cells after IL-1beta and compound C treated. ^∗∗^*P* < 0.01 versus sham group, ^##^*P* < 0.01 IL-1beta group versus IL-1beta + compound C group.

As reported that IL-1beta which could be secreted to extracellular from microglia/macrophages was the major cytokine after NLRP3 was activated [33]. PC12 cell was a kind of neuron line which was widely used in vitro experiments. Here, IL-1beta was used to stimulate PC12 cells to investigate our previously proved conclusion in vivo. Compound C is a kind of autophagy inhibitor that plays function by the AMPK/Beclin-1 pathway. First, IL-1beta with a dosage of 10 *μ*g/l was used to stimulate PC12 cells, and we found more Atg5 and fewer p62 positive neurons compared with the PBS-treated group. Next, Atg5 and p62 positive neurons showed an opposite change trend after compound C was added to inhibit autophagy (Figure 6(a)). In addition, the expression of LC3B and p62 which indicated the different processes of autophagy were measured with Western blots. The quantitative of autophagy proteins showed the same results as before (Figure 6(c)). Whether as the vivo experiment results that excessive autophagy-mediated neurons apoptosis, we used different ways to assess neurons function after different treatments. IL-1beta treatment mediated more neurons apoptosis compared with the PBS treated group both in TUNEL-staining and flow cytometry. When inhibiting autophagy with compound C, the percentage of apoptosis neurons decreased after IL-1beta treatment (Figures 6(b) and 6(d)). The vitro experiment results also supported our vivo experiment with that excessive autophagy-mediated neurons apoptosis.

## 4. Discussion

**Figure 7:**
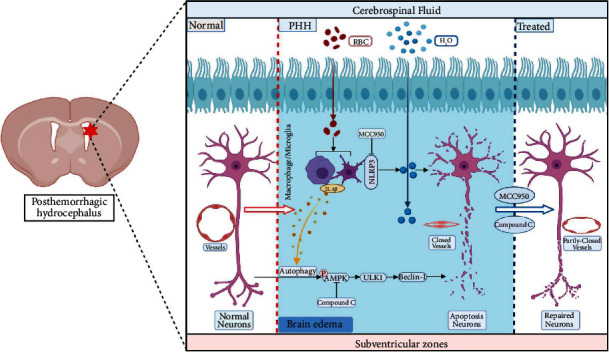
Schematic mechanism of NLRP3 activation in microglia/macrophages contributes to subependymal edema and neurons apoptosis by upregulating autophagy through AMPK/ULK1/Beclin-1 pathway. After ICH-IVH, rats occurred subependymal edema which contributes to cognitive dysfunction. Next, we found NLRP3 inflammasome activation in microglia/macrophage-mediated neurons excessive autophagy, and excessive autophagy caused neuron damage through the AMPK/Beclin-1 pathway. Administration NLRP3 specific inhibitor MCC950 could reduce edema in SVZ and improve neurofunction after hydrocephalus.

In this study, we found that activation of NLRP3 in microglia/macrophages contributes to subependymal edema formation and cognitive dysfunction after ICH-IVH, and the edema was nonvascular origin. NLRP3 inflammasome aggravated neuron apoptosis by upregulating autophagy through the AMPK/Beclin-1 pathway in SVZ after ICH-IVH (Figure 7; this image is plotted by Biorender). Besides, IL-1beta whose secretion was mainly promoted by NLRP3 activation mediated autophagy-induced PC12 cells apoptosis. This study provides compelling evidence that NLRP3 activation mediated edema formation and neuron apoptosis in SVZ played a pivotal role in the pathogenesis of PHH.

After ICH, resident glial cells were activated and circulating immune cells were recruited to participate in the occurrence and development of neuroinflammation [34–36]. Microglia was the resident immune cell distributed in brain that could be rapidly activated to mediate neuroinflammation in response to pathological conditions, including hypoxia, infection, and brain tissue injury [37]. Besides directly attacking neurons, the polarized microglia could also indirectly damage the neurons by changing the microenvironment via releasing neurotoxicity factors such as IL-1beta or recruiting other neurotoxicity cells such as macrophages to amplify inflammatory response [38]. Inflammation cytokines (IL-1beta, IL-6, IL-27, and TNF-*α*) mainly released from microglia/macrophages and neutrophils during neuroinflammation aggravated secondary injury to neurons [16, 39, 40]. Thus, inhibiting neuroinflammation might play a protective role in hydrocephalus after hemorrhage.

Nucleotide-binding oligomerization domain-like receptor containing pyrin domain 3 (NLRP3) inflammasome, the apoptosis-associated speck-like protein, has been proposed as a crucial mediator in innate immunity [41]. The activated NLRP3 could cleave pro-IL-1beta and pro-IL-18 into their mature and functional form, resulting in the activation of a subsequent inflammatory response [41, 42]. Recent studies indicated NLRP3 inflammasome could also polarize microglia and exacerbate ischemia/hemorrhage-induced brain injury [33, 43]. NLRP3 activation aggravates neuronal cell death and behavioral deficits, knockdown or downregulating NLRP3 could improve neurological functions of ICH animals [44]. In addition, cell swelling, edema and inflammation are closely related. It has been proved that edema could cause neuron dysfunction, and most of studies considered that BBB dysfunction is the main reason for brain edema [45, 46]. In this study, we found that rats showed obvious subependymal edema which was nonvascular origin, while NLRP3 was widely activated in microglia/macrophages on day 3 after ICH-IVH. In addition, we found that using NLRP3 inhibitor MCC950 could reduce subependymal edema and improve the cognitive function of ICH-IVH rats. Therefore, we aim at neurons to explore how the NLRP3 activation in microglia/macrophages influences cognitive and motor function after hemorrhage.

As the terminally differentiated cells, neurons did not divide and replicate themselves, which was the main reason a severe brain injury was often difficult to recover [47]. Autophagy was a conserved intracellular process to degrade dysfunctional organelles and protein aggregates and played an essential role in maintaining neuronal homeostasis [47]. Much of the evidence to support this derives from studies adjusting autophagy in neurons and observing neurodegeneration, especially in AD and PD [48–50]. Normal autophagy was considered as a protective factor against neurodegeneration, infection, and brain injury disease [51]; however, autophagy dysfunction was associated with increased susceptibility of neurons to ischemic injury. It was reported that the activation of autophagy was coincide with axonal swelling of PC12 cells when nerve growth factor was deprived or cells were in excitotoxicity, suggesting a close relationship between autophagic process and neurite degeneration [52]. In addition, neurotoxin exposure induced apoptosis with a concomitant increase of autophagy flux in primary cortical neurons [53]. All evidence indicated that autophagy flux participated in neuronal injury in many kinds of CNS diseases. However, the role of autophagy after ICH remained controversial. Studies found that autophagy-related disorders promoted the occurrence of stroke in some cases [54], and autophagy exacerbated brain injury after ICH. Autophagic cell death of neurons after ICH was confirmed by using conditional knockout Atg7 mice [55]. Although autophagy was involved in promoting brain injury during the acute phase of ICH, studies showed that autophagy had a neuroprotective function via clearing up the accumulation of cell rubbish [56], and the antineuronal apoptosis effects were related to the enhancement of autophagy [57]. Several studies showed that ICH induced autophagy of immune cells, especially microglia/macrophages, contributing to the improvement of outcomes by regulating inflammation [58–60]. Hence, we aimed to explore the relationship between neuronal apoptosis and NLRP3-mediated autophagy in the acute phase of ICH-IVH and to prove that excessive neuronal autophagy aggravated neuronal apoptosis, which contributed to cognitive dysfunction after ICH-IVH.

NLRP3 activation in microglia/macrophages was the critical process to adjust neuronal autophagy, and according to the RNA sequencing results, we found that NLRP3 mediated neuronal autophagy through the AMPK/Beclin-1 pathway. Since NLRP3 activation could promote the release of cytokines, among which IL-1beta played the most important role among these factors [61, 62], we further explored the role of IL-1beta and found that IL-1beta was the key factor mediating excessive autophagy and neuronal apoptosis after NLRP3 activation by using PC12 cells in vitro.

Several limitations need to be mentioned in this study. First, NLRP3 plays multifunctional roles in inflammation response, and NLRP3 inflammasome is activated in many kinds of neural cells. Further research is needed to investigate the other mechanisms underlying the neuroprotective effects of NLRP3 inhibition in secondary brain injury after ICH-IVH. In addition, since NLRP3 is not only expressed on microglia/macrophages as our immunofluorescence staining showed, further study about the roles of NLRP3 on other CNS cells, such as astrocytes, is necessary. Second, we found that NLRP3 activation could adjust subependymal edema formation, but the specific mechanism NLRP3 mediated cellular, and nonangiogenic interstitial edema still needs investigation. Third, how the neuron autophagy affects neuronal function after hemorrhage and the related mechanism associated with NLRP3 should be more deeply evaluated. Recent studies and our present study have demonstrated the effectiveness of inhibiting NLRP3 expression by using MCC950 [63, 64]. However, the time window and stability of MCC950 are very limited. This study only focused on the early pathophysiological changes (3 days) in SVZ after ICH-IVH, the longer time research is needed in the further study. At the same time, the effects of NLRP3 on specific cell types require more precise gene-editing techniques, such as the use of cre/loxp rat to edit NLRP3 genes in specific cells.

## 5. Conclusion

An earlier version of this work has been present as preprint in Research Square [65]. The present study demonstrated that NLRP3 inflammasome activation in microglia/macrophages aggravated edema formation and neuronal apoptosis after ICH-IVH in SVZ, and neuron apoptosis was mediated by upregulating autophagy through AMPK/Beclin-1 signaling pathway. At least in part, NLRP3-related extracellular cytokine IL-1beta contributed to this process. Therefore, inhibition NLRP3 activation may be a potential therapeutic strategy which could reduce subependymal edema to improve cognitive function in the management of hydrocephalus patients after hemorrhage.

## Data Availability

All data generated or analyzed during this study are included in this published article. The datasets used and/or analyzed during the current study are available from the corresponding author on reasonable request.

## Abbreviations

AD: Alzheimer disease
Atg5: Autophagy related 5 homolog
AMPK: Adenosine 5′-monophosphate- (AMP-) activated protein kinase
FJC: Fluoro-jade C
Iba1: Ionized calcium-binding adaptor molecule 1
ICH: Intracerebral hemorrhage
ICH-IVH: Intracerebral hemorrhage with ventricle extension
IL-1beta: Interleukin-1beta
IL-18: Interleukin-18
IL-6: Interleukin-6
IL-27: Interleukin-27
LC3B: Microtubule-associated proteins 1A/1B light chain 3B
NLRP3: NACHT, LRR and PYD domains-containing protein 3
p-AMPK: Phospho-adenosine 5′-monophosphate- (AMP-) activated protein kinase
PBS: Phosphate-buffered saline
PD: Parkinson disease
PHH: Posthemorrhagic hydrocephalus
p62: Sequestosome-1
SVZ: Subventricular zone
TNF-*α*: Tumor necrosis factor-*α*
TUNEL: Terminal deoxynucleotidyl transferase dUTP nick end labeling
ULK1: Serine/threonine-protein kinase ULK1
ZO-1: Zonula occludens 1.

## Supplementary Materials

Supplementary Materials Figure S1: GO and KEEG enrichment results according to transcriptional analysis. (a, c) GO enrichment showed different expression genes clustered into cellular function after ICH-IVH and MCC950 treatment in SVZ. (b, d) KEEG enrichment clustered different expression genes into respective pathways.
