# Supplementary material for: Inhibiting Microglia-Derived NLRP3 Alleviates Subependymal Edema and Cognitive Dysfunction in Posthemorrhagic Hydrocephalus after Intracerebral Hemorrhage via AMPK/Beclin-1 Pathway
Source: Oxid Med Cell Longev. 2022 May 17;2022:4177317. doi: 10.1155/2022/4177317 (PMC9129981; doi:10.1155/2022/4177317)
Supplement: Supplementary Materials — Figure S1: GO and KEEG enrichment results according to transcriptional analysis. (a, c) GO enrichment showed different expression genes clustered into cellular function after ICH-IVH and MCC950 treatment in SVZ. (b, d) KEEG enrichment clustered different expression genes into respective pathways. [file 4177317.f1.pdf]

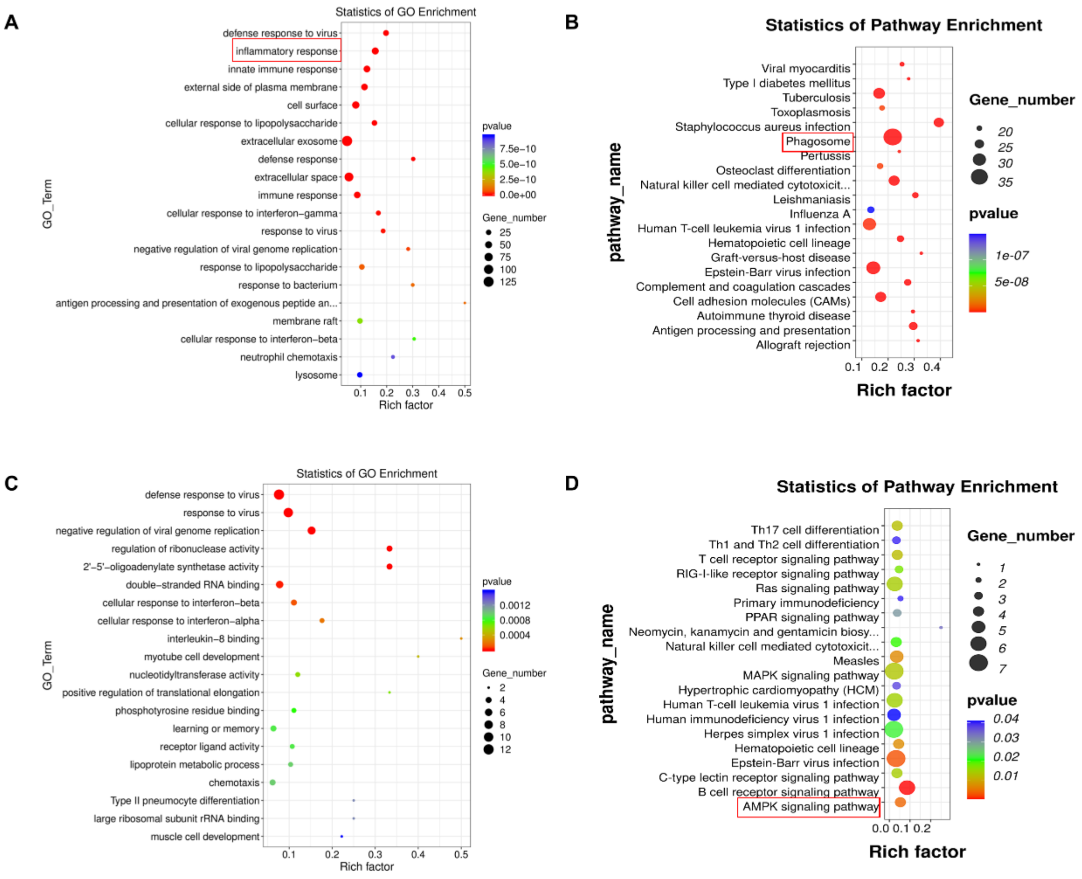

Figure S1. GO and KEEG enrichment results according to transcriptional analysis.

(A and C) GO enrichment showed different expression genes clustered into cellular function after ICH-IVH and MCC950 treatment in SVZ. (B and D) KEEG enrichment clustered different expression genes into respective pathways.
